# Multicenter Validation Study of the Clinical Diagnostic Criteria for IgG4‐Related Sclerosing Cholangitis 2020 in Japan

**DOI:** 10.1002/jhbp.70056

**Published:** 2026-01-07

**Authors:** Itaru Naitoh, Takahiro Nakazawa, Kensuke Kubota, Takayoshi Nishino, Akira Nakamura, Dai Inoue, Takanori Sano, Kazuhiro Kikuta, Yusuke Kurita, Kazuro Chiba, Tsukasa Ikeura, Hiroyuki Matsubayashi, Takuya Ishikawa, Masaki Kuwatani, Terumi Kamisawa, Ichiro Yasuda, Mitsuhiro Kawano, Atsushi Masamune, Japan Pancreatitis Study Group, Yoko Abe, Atsushi Irisawa, Hiroyuki Tanaka, Eizaburo Ohno, Tatsuru Maruo, Toshiharu Ueki, Rei Suzuki, Tadayuki Takagi, Hiromasa Ohira, Shirou Tanoue, Akio Ido, Takahiro Komori, Kayashima Atsuo, Masayasu Horibe, Eisuke Iwasaki, Masahiro Tsujimae, Yuzo Kodama, Kazushige Uchida, Masayuki Ueno, Kenji Notohara, Shotaro Kakehashi, Nao Fujimori, Eiji Nakatani, Yasuki Hori, Michihiro Yoshida, Hiroki Kawashima, Kazunao Hayashi, Shuji Terai, Kosaku Morimoto, Ryosuke Sato, Kazuyuki Matsumoto, Hironari Kato, Yoshiharu Masaki, Hiroshi Nakase, Toshihiko Arizumi, Atsushi Tanaka, Ryotaro Matusmoto, Tetsuya Takikawa, Ryosuke Tonozuka, Takao Itoi, Kenji Hirano, Nobuhiko Hayashi

**Affiliations:** ^1^ Department of Gastroenterology Nagoya City University Midori Municipal Hospital Nagoya Japan; ^2^ Department of Gastroenterology and Metabolism Nagoya City University Graduate School of Medical Sciences Nagoya Japan; ^3^ Department of Gastroenterology and Hepatology Yokohama City University Hospital Yokohama Japan; ^4^ Department of Gastroenterology Tokyo Women's Medical University Yachiyo Medical Center Yachiyo Japan; ^5^ Department of Medicine Gastroenterology, Shinshu University Matsumoto Nagano Japan; ^6^ Department of Radiology Kanazawa University Hospital Kanazawa Japan; ^7^ Division of Gastroenterology Tohoku University Graduate School of Medicine Sendai Japan; ^8^ Department of Internal Medicine Tokyo Metropolitan Komagome Hospital Tokyo Japan; ^9^ Third Department of Internal Medicine, Division of Gastroenterology and Hepatology Kansai Medical University Hirakata Japan; ^10^ Division of Endoscopy Shizuoka Cancer Center Shizuoka Japan; ^11^ Department of Gastroenterology and Hepatology Nagoya University Graduate School of Medicine Nagoya Japan; ^12^ Department of Gastroenterology and Hepatology Hokkaido University Hospital Sapporo Japan; ^13^ Division of Gastroenterology and Center of IgG4‐Related Disease, Department of Internal Medicine Tokyo Metropolitan Komagome Hospital Tokyo Japan; ^14^ Third Department of Internal Medicine University of Toyama Toyama Japan; ^15^ Department of Hematology & Immunology Kanazawa Medical University Kahoku‐Gun Japan

**Keywords:** autoimmune pancreatitis, cholangiocarcinoma, IgG4‐related disease, IgG4‐related sclerosing cholangitis, primary sclerosing cholangitis

## Abstract

**Background:**

The diagnostic performance of clinical diagnostic criteria for IgG4‐related sclerosing cholangitis 2020 (IgG4‐SC2020) has not been fully validated since its proposal as a revision of the 2012 criteria (IgG4‐SC2012).

**Methods:**

We conducted a multicenter validation study to evaluate the diagnostic performance of IgG4‐SC2020 using clinical data collected from 1034 patients with IgG4‐SC and 447 patients with mimickers, including 143 with pancreatic cancer, 157 with primary sclerosing cholangitis, and 147 with cholangiocarcinoma in Japan.

**Results:**

The sensitivity of IgG4‐SC2020 was significantly higher than that of IgG4‐SC2012 (99.0% vs. 89.1%; *p* < 0.001). The specificities of both IgG4‐SC2020 and IgG4‐SC2012 were 100% for pancreatic cancer and cholangiocarcinoma. For primary sclerosing cholangitis, the specificities of IgG4‐SC2020 and IgG4‐SC2012 were 97.5% and 100%, respectively, with no significant difference (*p* = 0.123). A total of 113 patients who could not be diagnosed according to the IgG4‐SC2012 were successfully diagnosed using IgG4‐SC2020. These diagnostic improvements were attributed to the inclusion of MRCP findings (*n* = 97), the absence of neoplastic cells on histology (*n* = 15), and the presence of IgG4‐related kidney lesions (*n* = 1).

**Conclusions:**

This Japanese multicenter validation study demonstrated that the diagnostic performance of IgG4‐SC2020 was superior to that of IgG4‐SC2012.

**Trial Registration:** UMIN Clinical Trials Registry (UMIN‐CTR), UMIN000052984.

## Graphical abstract

In this Japanese multicenter validation study, Naitoh and colleagues analyzed clinical data from 1034 patients with IgG4‐related sclerosing cholangitis to evaluate the 2020 clinical diagnostic criteria. The IgG4‐SC2020 criteria demonstrated higher sensitivity than the 2012 criteria while maintaining high specificity, and reclassified cases not diagnosable under the 2012 criteria.

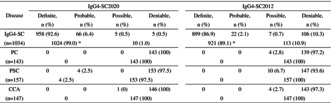

**Abbreviations**
AIP: autoimmune pancreatitis
CCA: cholangiocarcinoma
ERC: endoscopic retrograde cholangiography
ERCP: endoscopic retrograde cholangiopancreatography
EUS: endoscopic ultrasonography
IDUS: intraductal ultrasonography
IgG4‐RD: IgG4‐related disease
IgG4‐SC: IgG4‐related sclerosing cholangitis
MRCP: magnetic resonance cholangiopancreatography
PC: pancreatic cancer
PSC: primary sclerosing cholangitis

## 1 Introduction

IgG4‐related sclerosing cholangitis (IgG4‐SC) is a distinct type of sclerosing cholangitis, characterized by elevated serum IgG4 levels, marked lymphoplasmacytic infiltration, fibrosis with abundant IgG4‐positive plasma cells, and a favorable response to steroid therapy [1, 2]. It is often associated with autoimmune pancreatitis (AIP) and is recognized as a bile duct manifestation of IgG4‐related diseases (IgG4‐RD) [3, 4]. Epidemiological features, clinical characteristics, and long‐term outcomes of IgG4‐SC have been reported in nationwide Japanese surveys conducted in 2012, 2015, and 2019 [5, 6, 7, 8]. IgG4‐SC exhibits diverse cholangiographic patterns, and pancreatic cancer (PC), primary sclerosing cholangitis (PSC), and cholangiocarcinoma (CCA) are important mimickers because of their similar cholangiographic features.

Two sets of diagnostic criteria for IgG4‐SC have been proposed: the “HISORt” criteria in the United States [9] and the Clinical Diagnostic Criteria for IgG4‐SC 2012 (IgG4‐SC2012) in Japan (Table S1) [10]. In 2020, the IgG4‐SC2012 criteria were revised to the Clinical Diagnostic Criteria 2020 (IgG4‐SC2020) to improve diagnostic performance (Table S2) [2]. The major revisions in IgG4‐SC2020 were as follows: (i) thickening of the bile duct wall was added as a separate diagnostic item, in addition to bile duct narrowing; (ii) magnetic resonance cholangiopancreatography (MRCP) was incorporated for the assessment of bile duct narrowing; (iii) negative findings for malignancy on bile duct biopsy were included in the pathological criteria; and (iv) kidney lesions were added as other organ involvement, in accordance with the International Consensus Diagnostic Criteria [11] and the Clinical Diagnostic Criteria for AIP 2018 proposed by the Japan Pancreas Society (JPS2018) [12]. IgG4‐SC2020 consists of six criteria: narrowing of the intrahepatic and/or extrahepatic bile ducts, thickening of the bile duct wall, serological findings, pathological findings, coexistence with other IgG4‐RDs, and response to steroid therapy. Since its introduction, IgG4‐SC2020 has been utilized in clinical practice for the diagnosis of IgG4‐SC; however, its diagnostic performance has not yet been fully validated, even after several years of use. We therefore conducted a multicenter validation study in Japan to evaluate the diagnostic performance of IgG4‐SC2020 using clinical data collected from patients with IgG4‐SC and its mimickers.

## 2 Methods

### 2.1 Patients

Clinical information of patients with IgG4‐SC, PC, PSC, and CCA was collected through the All‐Japan Research Group for Establishing Diagnostic Criteria and Clinical Practice Guidelines for IgG4‐related Disease, Research Program for Intractable Disease, under the support of the Ministry of Health, Labor and Welfare of Japan (Chairperson: Mitsuhiro Kawano) and the Subcommittee of the Pancreatitis Research Committee, the Japan Pancreas Society (the Japan Pancreatitis Study Group for AIP). All patients were diagnosed with IgG4‐SC, PC, PSC, or CCA after 2000.

Definite or probable IgG4‐SC was diagnosed according to the IgG4‐SC2012 or IgG4‐SC2020 criteria. We defined the cases classified as definite or probable diagnoses based on the respective diagnostic criteria as IgG4‐SC. Definite or probable PSC was diagnosed based on the 2016 diagnostic criteria for PSC [13]. PC and CCA were pathologically confirmed as malignant using biopsy or surgical specimens. Serum IgG4 levels were measured in all patients with PC, PSC, and CCA.

### 2.2 Data Collection and Analysis

We collected clinical data on IgG4‐SC cases for the central analysis at Nagoya City University Midori Municipal Hospital from participating institutions across Japan. All IgG4‐SC cases were independently evaluated by local investigators at each participating institution according to both the 2012 and 2020 criteria. The clinical data included age, sex, serum IgG4 level, cholangiographic findings (type 1, type 2, type 3, type 4, or unclassified, according to the cholangiographic classification of IgG4‐SC [1, 2]), narrowing of the bile duct, thickening of the bile‐duct wall, pathological findings, specimen for pathological analysis (biopsy or surgical specimen), other organ involvement (AIP, IgG4‐SC, sclerosing dacryoadenitis/sialadenitis, retroperitoneal fibrosis, or kidney lesion), treatment (steroid administration or surgery), and effectiveness of steroid therapy. Surgery was defined as cases in which pathological findings were obtained from surgical specimens. Clinical data on diagnostic items and diagnoses according to the IgG4‐SC2012 and IgG4‐SC2020 were also collected. Imaging and histopathological evaluations were independently assessed by local investigators at each participating institution. Assessment of IgG4‐SC2012 and IgG4‐SC2020 was also analyzed by local investigators.

In the central analysis at Nagoya City University Midori Municipal Hospital, we evaluated the diagnosis (definite, probable, possible, or deniable) according to the IgG4‐SC2020 and IgG4‐SC2012, as well as the diagnostic yields (sensitivity and specificity) of both criteria for IgG4‐SC. Diagnostic accuracy was also analyzed before surgery as preoperative diagnostic accuracy. Furthermore, we investigated diagnostic changes between IgG4‐SC2020 and IgG4‐SC2012 and assessed the reasons for these changes.

This study was approved by the institutional review board of the lead institution (Nagoya City University Midori Municipal Hospital; approval number: 60‐23‐0100) and by the institutional review boards of all participating institutions. It was conducted in accordance with the guidelines of the Declaration of Helsinki (Clinical Trial Registration Number: UMIN000052984).

### 2.3 Statistical Analysis

The number of mimicker (PC, PSC, and CCA) cases was determined using a precision‐based sample size calculation. Assuming that no false‐positive results would be observed among 135 non‐diseased cases, the one‐sided 95% lower confidence limit of specificity, estimated by the Clopper‐Pearson method [14], would be approximately 97.8%. Therefore, a total of 135 mimicker cases was considered sufficient to ensure high specificity with adequate precision.

Statistical comparisons were made using Pearson's chi‐square test or Fisher's exact test for categorical variables, and the Mann–Whitney *U* test for continuous variables. All statistical analyses were performed with EZR (version 1.42; Saitama Medical Center, Jichi Medical University, Saitama, Japan). A *p* value < 0.05 was considered statistically significant.

## 3 Results

### 3.1 Characteristics of the Enrolled Patients

A total of 1034 patients with IgG4‐SC were enrolled from 28 institutions across Japan. In addition, 143 patients with PC, 157 with PSC, and 147 with CCA were enrolled as disease mimickers of IgG4‐SC. The characteristics of these patients are summarized in Table 1. The proportion of male patients was higher in IgG4‐SC than PC (*p* < 0.001), PSC (*p* < 0.001), and CCA (*p* = 0.008). Patients with IgG4‐SC were significantly older than those with PSC (*p* < 0.001), but significantly younger than those with PC (*p* < 0.001) and CCA (*p* < 0.001). Serum IgG4 levels were higher in patients with IgG4‐SC than those with mimickers (all; *p* < 0.001). Elevated serum IgG4 levels (≥ 135 mg/dL) were observed in 873 out of 1002 patients (87.1%) with IgG4‐SC in whom serum IgG4 was measured, whereas the elevated serum IgG4 levels were observed only in 7.0% of patients with PC, 6.4% with PSC, and 6.1% with CCA (all; *p* < 0.001). Cholangiographic classification of IgG4‐SC was type 1 in 672 patients (65.0%), type 2 in 154 (14.9%), type 3 in 98 (9.5%), type 4 in 96 (9.3%), and unclassified in 14 (0.1%). The most frequent cholangiographic types were type 1 in PC, type 2 in PSC, and type 4 in CCA. Surgery was performed in 28 patients with IgG4‐SC, 49 with PC, 10 with PSC, and 71 with CCA. Surgery was performed less frequently in patients with IgG4‐SC than in those with PC (*p* < 0.001), PSC (*p* = 0.015), or CCA (*p* < 0.001). Surgery includes complete resections of the lesion in cases of IgG4‐SC, PC, PSC, and CCA when malignancy is suspected, as well as liver transplantation in PSC.

**TABLE 1:** Characteristics of the enrolled patients.

	IgG4‐SC	Mimickers		
		PC	PSC	CCA
Number of patients	1034	143	157	147
Sex, male, *n* (%)	824 (79.7)	78 (54.5)	90 (57.3)	103 (70.0)
Age, years, median (IQR)	69 (61–75)	74 (66–78)	44 (26–62)	73 (68–77)
Serum IgG4 level, mg/dL, median (IQR)	363 (189–731)	35 (21–58)	40 (23–73)	42 (22–72)
Serum IgG4 level ≥ 135 mg/dL, *n* (%)	873 (87.1)^a^	10 (7.0)	10 (6.4)	9 (6.1)
Cholangiogram (Type 1: 2: 3: 4: unclassified)	672: 154: 98: 96: 14	139: 1: 0: 0: 3	0: 126: 9: 11: 11	1: 1: 1: 132: 12
Steroid administration, *n* (%)	847 (81.9)	0 (0)	5 (3.2)	6 (4.1)
Steroid effectiveness, (%)	841/847 (99.3)	0 (0)	0/5 (0)	0/6 (0)
Surgery, *n* (%)	28 (2.7)	49 (34.3)	10 (6.4)	71 (48.3)

*Note:* Surgery: complete resection in IgG4‐SC, PC, PSC, and CCA, liver transplantation in PSC.

Abbreviations: CCA, cholangiocarcinoma; IgG4‐SC, IgG4‐related sclerosing cholangitis; IQR, interquartile range; PC, pancreatic cancer; PSC, primary sclerosing cholangitis.

^a^ Out of 1002 patients.

### 3.2 Fulfillment of Diagnostic Items on IgG4‐SC2020 and IgG4‐SC2012

Fulfillment of each diagnostic criterion of IgG4‐SC2020 and IgG4‐SC2012 among patients with IgG4‐SC is shown in Table 2. In IgG4‐SC2020, narrowing of the intrahepatic and/or extrahepatic bile duct (I) was fulfilled in all patients on endoscopic retrograde cholangiography (ERC) (Ia) in 937 patients (9.6%) or on MRCP (Ib) in 97 patients (9.4%). Because MRCP was not included for the narrowing of bile duct in IgG4‐SC2012, these 97 cases did not meet the diagnostic criterion (I). Thickening of the bile‐duct wall (II) was observed on 1030 out of 1034 patients (99.6%): on endoscopic ultrasonography (EUS) or intraductal ultrasonography (IDUS) (IIa) in 763 patients (73.8%) and on computed tomography, magnetic resonance imaging, or ultrasonography (IIb) in 267 patients (25.8%). Regarding serological findings (III), serum IgG4 was measured in 1002 out of 1034 patients (96.9%), and 873 patients (87.1%) had serum IgG4 level of ≥ 135 mg/dL. Regarding pathological findings (IV), IVa, IVb, and IVc were identified in 95 patients (9.2%), 532 patients (51.4%), and 54 patients (5.2%), respectively. Regarding other organ involvement (V), AIP (Va) was detected in 945 patients (91.3%), IgG4‐RD in other organs (IgG4‐related dacryoadenitis/sialadenitis, IgG4‐related retroperitoneal fibrosis, IgG4‐related kidney lesion [Vb]) were identified in 22 patients (2.1%). Regarding steroid therapy (VI), steroids were effective in 841 patients (81.3%) and ineffective in 6 patients (0.6%).

**TABLE 2:** Fulfillment of each diagnostic criterion in IgG4‐SC 2020 and 2012.

IgG4‐SC2020	I	a (ERC): b (MRCP)	937: 97
	II	a (EUS/IDUS): b (CT/MRI/US): Not fulfilled	763: 267: 4
	III	Serum IgG4 ≥ 135 mg/dL: Not fulfilled: Not applicable	873: 129: 32
	IV	a: b: c: Not applicable	95: 532: 54: 353
	V	a (AIP): b (Other IgG4‐RD): Not fulfilled	945: 22: 67
	VI	Steroid effectiveness: Not fulfilled: Not applicable	841: 6: 187
IgG4‐SC2012	I	Fulfilled (ERC): Not applicable	937: 97
	II	Serum IgG4 ≥ 135 mg/dL: Not fulfilled: Not applicable	873: 129: 32
	III	Other organ involvement: Not fulfilled	966: 68
	IV	a + b + c/d: a + b: Not fulfilled: Not applicable	53: 91: 545: 345
	Option	Steroid effectiveness: Not fulfilled: Not applicable	841: 6: 187

Abbreviations: AIP, autoimmune pancreatitis; CT, computed tomography; ERC, endoscopic ultrasonography; EUS, endoscopic ultrasonography; IDUS, intraductal ultrasonography; IgG4‐RD, IgG4‐related disease; IgG4‐SC2012, clinical diagnostic criteria for IgG4‐SC 2012; IgG4‐SC2020, clinical diagnostic criteria for IgG4‐SC 2020; MRCP, magnetic resonance cholangiopancreatography; MRI, magnetic resonance imaging; US, ultrasonography.

### 3.3 Comparison of Diagnostic Performance Between IgG4‐SC2020 and IgG4‐SC2012

We then compared the diagnostic performance between IgG4‐SC2012 and IgG4‐SC2020 in the overall cohort, including the subset of patients who underwent surgery and for whom pathological findings were obtained from surgical specimens. According to the IgG4‐SC2020, final diagnoses were definite in 958 patients (92.6%), probable in 66 (6.4%), possible in 5 (0.6%), and deniable in 5 (0.4%) (Table 3). According to the IgG4‐SC2012, final diagnoses were definite in 899 patients (86.9%), probable in 22 (2.1%), possible in 7 (0.7%), and deniable in 106 (10.3%). If we defined the cases classified as definite or probable diagnoses as IgG4‐SC, the sensitivity was 99.0% for IgG4‐SC2020 and 89.1% for IgG4‐SC2012. The sensitivity was significantly higher in IgG4‐SC2020 than in IgG4‐SC2012 (*p* < 0.001). No patients with PC or CCA were diagnosed with IgG4‐SC according to the IgG4‐SC2020 or IgG4‐SC2012, indicating a specificity of 100%. Four patients with PSC were diagnosed as probable IgG4‐SC according to the IgG4‐SC2020, based on the combination of Ia + IIa + III + IVb (non‐neoplastic cells). They were diagnosed as possible IgG4‐SC according to the IgG4‐SC2012, based on the combination of (1) bile duct imaging + (2) elevated serum IgG4 level. Thus, the specificity in patients with PSC was 97.5%, according to the IgG4‐SC2020 and 100% according to the IgG4‐SC2012, with no significant difference between them (*p* = 0.123).

**TABLE 3:** Comparison of diagnostic performance between IgG4‐SC2020 and IgG4‐SC2012.

Disease	IgG4‐SC2020				IgG4‐SC2012			
	Definite, *n* (%)	Probable, *n* (%)	Possible, *n* (%)	Deniable, *n* (%)	Definite, *n* (%)	Probable, *n* (%)	Possible, *n* (%)	Deniable, *n* (%)
IgG4‐SC (*n* = 1034)	958 (92.6)	66 (6.4)	5 (0.5)	5 (0.5)	899 (86.9)	22 (2.1)	7 (0.7)	106 (10.3)
	1024 (99.0) *		10 (1.0)		921 (89.1) *		113 (10.9)	
PC (*n* = 143)	0	0	0	143 (100)	0	0	4 (2.8)	139 (97.2)
	0		143 (100)		0		143 (100)	
PSC (*n* = 157)	0	4 (2.5)	0	153 (97.5)	0	0	10 (6.7)	147 (93.6)
	4 (2.5)		153 (97.5)		0		157 (100)	
CCA (*n* = 147)	0	0	1 (0)	146 (100)	0	0	4 (2.7)	143 (97.3)
	0		147 (100)		0		147 (100)	

Abbreviations: CCA, cholangiocarcinoma; IgG4‐SC, IgG4‐realated sclerosing cholangitis; IgG4‐SC2012, clinical diagnostic criteria for IgG4‐SC 2012; IgG4‐SC2020, clinical diagnostic criteria for IgG4‐SC 2020; PC, pancreatic cancer; PSC, primary sclerosing cholangitis.

* *p* < 0.001 between IgG4‐SC2020 and IgG4‐SC2012.

### 3.4 Cases Whose Diagnosis Was Different According to the IgG4‐SC2020 and IgG4‐SC2012

**FIGURE 1:**
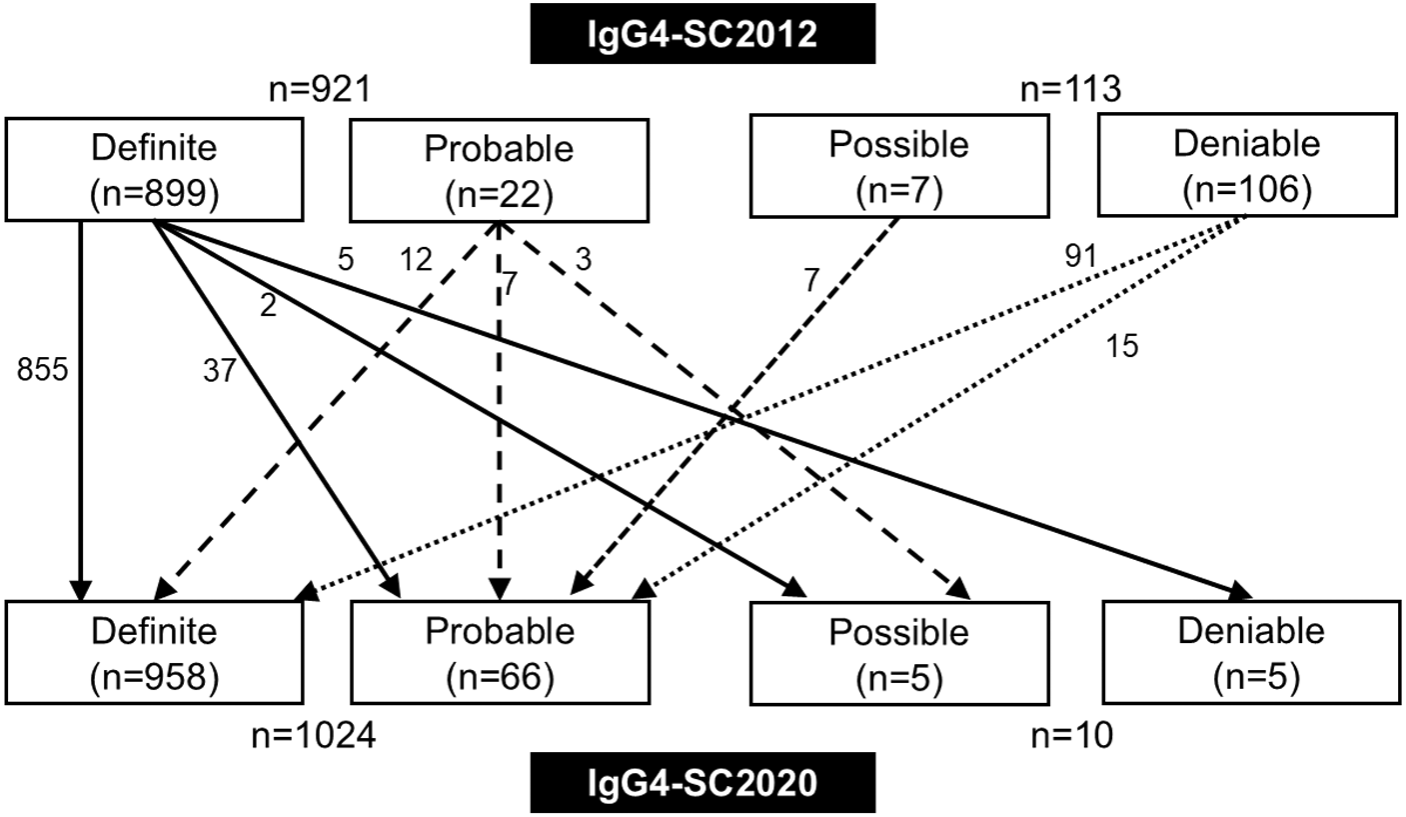
Change in diagnosis according to the IgG4‐SC 2012 and 2020 criteria.

Among the 899 cases with definite IgG4‐SC according to the IgG4‐SC2012, the diagnosis remained unchanged in 855 and was downgraded in 44 (to probable in 37, possible in 2, and deniable in 5) according to the IgG4‐SC2020 (Figure 1). Among the 22 cases with probable IgG4‐SC, the diagnosis remained unchanged in 7, was upgraded to definite IgG4‐SC in 12, and downgraded to possible IgG4‐SC in 3. Collectively, among the 921 cases with IgG4‐SC, the diagnosis of IgG4‐SC remained unchanged in 911 and was downgraded in 10 (to probable in 5 and to deniable in 5). The clinical characteristics of these 10 downgraded cases are presented in Table 4. Three patients could not be diagnosed as having IgG4‐SC because thickening of the bile duct wall (II) could not be detected.

**TABLE 4:** Clinical characteristics of 10 patients with IgG4‐SC downgraded from IgG4‐SC2012 to IgG4‐SC2020.

Case#	Cholangiogram	IgG4‐SC2020							IgG4‐SC2012					
		Diagnosis	I	II	III	IV	V	VI	Diagnosis	I	II	III	IV	Option
1	1	Deniable	a	×	○	N/A	a	○	Definite	○	○	○	N/A	○
2	1	Deniable	a	×	○	b	a	○	Definite	○	○	○	×	○
3	2	Deniable	a	×	○	N/A	a	○	Definite	○	○	○	N/A	○
4	3	Deniable	a	b	×	a	a	N/A	Definite	○	×	○	a, b	N/A
5	3	Deniable	a	b	×	b	a	N/A	Definite	○	×	○	×	N/A
6	4	Possible	a	b	○	N/A	a	N/A	Definite	○	○	○	N/A	N/A
7	4	Possible	a	b	○	N/A	×	○	Probable	○	○	×	N/A	○
8	4	Possible	a	b	○	b	×	○	Probable	○	○	×	×	○
9	Unclassified	Possible	a	b	○	N/A	×	○	Probable	○	○	×	N/A	○
10	Unclassified	Possible	a	b	○	N/A	b	○	Probable	○	○	○	N/A	○

Abbreviations: IgG4‐SC, IgG4‐realated sclerosing cholangitis; IgG4‐SC2020, clinical diagnostic criteria for IgG4‐SC 2020; IgG4‐SC2012, clinical diagnostic criteria for IgG4‐SC 2012; N/A, not applicable.

On the other hand, the diagnoses of 113 cases (7 possible and 106 deniable cases) were upgraded to definite in 91 or probable in 22 cases according to the IgG4‐SC2020. The diagnosis was upgraded based on MRCP findings showing bile duct narrowing in 97 patients. Among these 97 patients, serum IgG4 levels were elevated in 88 (out of 95 patients who underwent measurement; 92.6%), 69 (71.1%) had type 1 cholangiogram, and 92 (94.8%) had AIP. The diagnosis was also upgraded because of the fulfillment of “(v) No neoplastic cells identified” in the IgG4‐SC2020 in 15 patients. Among these 15 patients, none had AIP; 8 patients met the Ia + IIa + IVa criteria, and 7 met the Ia + IIa + III/VIb criteria. In addition, the diagnosis was upgraded in one patient due to the presence of IgG4‐related kidney lesions.

### 3.5 Comparison of Preoperative Diagnostic Performance Between IgG4‐SC2020 and IgG4‐SC2012

The preoperative diagnoses of the 28 patients with IgG4‐SC according to the IgG4‐SC2020 were as follows: definite in 1 (3.6%), probable in 3 (10.7%), possible in 9 (32.1%), and deniable in 15 (53.6%). After surgery, the diagnoses of the two patients initially classified as probable IgG4‐SC were upgraded to definite IgG4‐SC (Figure S1). In addition, the diagnoses of eight possible and fourteen deniable cases were upgraded to definite IgG4‐SC, and that of one possible case was upgraded to probable IgG4‐SC. Overall, 23 of the 24 cases were newly diagnosed as IgG4‐SC based on the incorporation of pathological findings from the surgical specimens (Table S3). Thus, the sensitivity for the diagnosis of IgG4‐SC increased from 14.3% (4 of 28 cases) to 96.4% (27 of 28 cases) (*p* < 0.001). One patient who could not be diagnosed as having IgG4‐SC based on the IgG4‐SC2020 was diagnosed as definite IgG4‐SC according to the IgG4‐SC2012, based on the combination of criteria (1) and (3), both pre‐ and postoperatively. According to the IgG4‐SC2012, the preoperative and postoperative sensitivities for IgG4‐SC were 25.0% (7 of 28 cases) and 96.4% (27 of 28 cases), respectively (Figure S2). The preoperative diagnostic performance of IgG4‐SC2020 and IgG4‐SC2012 is summarized in Table 5. Overall, the preoperative sensitivities for IgG4‐SC according to the IgG4‐SC2020 and IgG4‐SC2012 were 96.8% and 88.1%, respectively (*p* < 0.001).

**TABLE 5:** Comparison of preoperative diagnostic performance between IgG4‐SC2020 and IgG4‐SC2012.

Disease	IgG4‐SC2020				IgG4‐SC2012			
	Definite, *n* (%)	Probable, *n* (%)	Possible, *n* (%)	Deniable, *n* (%)	Definite, *n* (%)	Probable, *n* (%)	Possible, *n* (%)	Deniable, *n* (%)
IgG4‐SC (*n* = 1034)	934 (90.3)	67 (6.5)	14 (1.4)	19 (1.8)	888 (85.9)	23 (2.2)	9 (0.9)	124 (12.0)
	1001 (96.8) *		33 (3.2)		911 (88.1) *		133 (12.9)	
PC (*n* = 143)	0	0	0	143 (100)	0	0	8 (5.6)	135 (94.4)
	0		143 (100)		0		143 (100)	
PSC (*n* = 157)	0	4 (2.5)	0	153 (97.5)	0	0	10 (6.7)	147 (93.6)
	4 (2.5)		153 (97.5)		0		157 (100)	
CCA (*n* = 147)	0	0	4 (2.7)	143 (97.3)	0	0	8 (5.4)	139 (94.6)
	0		147 (100)		0		147 (100)	

Abbreviations: CCA, cholangiocarcinoma; IgG4‐SC, IgG4‐realated sclerosing cholangitis; IgG4‐SC2012, clinical diagnostic criteria for IgG4‐SC 2012; IgG4‐SC2020, clinical diagnostic criteria for IgG4‐SC 2020; PC, pancreatic cancer; PSC, primary sclerosing cholangitis.

* *p* < 0.001 between IgG4‐SC2020 and IgG4‐SC2012.

Among the 28 patients with IgG4‐SC who underwent surgery, preoperative serum IgG4 levels were measured in 17 (60.7%), and elevated levels were observed in 4 (23.5%) of these 17 patients. Cholangiographic findings were classified as type 1 in 11 patients, type 4 in 11, type 3 in 3, type 2 in 2, and unclassified in 1. EUS or IDUS was performed in 12 patients (42.9%). Bile duct biopsies were performed in 16 patients (57.1%), and no neoplastic cells were identified in any of them. An association with AIP was observed in 6 patients (21.4%), and a steroid trial was conducted in one patient (3.6%). Details of the preoperative diagnoses according to the IgG4‐SC2020 in the 28 patients with IgG4‐SC who underwent surgery are summarized in Table 6.

**TABLE 6:** Details of diagnosis and diagnostic items of IgG4‐SC2020 in 28 patients with IgG4‐SC who underwent surgery.

Case#	Diagnosis		Age	Sex	IgG4	Cholangiogram	Pre‐operative diagnostic items on IgG4‐SC2020					
	Pre‐ope	Post‐ope					I	II	III	IV	V	VI
1	Definite	Definite	64	M	140	4	a	a	○	b	a	N/A
2	Probable	Probable	68	M	N/A	1	a	b	N/A	N/A	a	N/A
3	Probable	Definite	75	M	86	1	a	b	×	b	a	N/A
4	Probable	Definite	61	M	61	1	a	b	×	b	a	N/A
5	Possible	Definite	43	M	N/A	4	a	a	N/A	b	×	N/A
6	Possible	Definite	54	M	134	4	a	a	×	b	×	N/A
7	Possible	Definite	74	M	N/A	4	a	a	N/A	b	×	N/A
8	Possible	Definite	83	M	97	4	a	a	×	b	×	N/A
9	Possible	Definite	85	M	43	4	a	a	×	b	×	N/A
10	Possible	Definite	73	M	54	4	a	a	×	b	×	N/A
11	Possible	Definite	72	M	101	4	a	a	×	b	×	N/A
12	Possible	Definite	75	M	293	4	a	b	○	b	a	N/A
13	Possible	Probable	78	M	65	4	a	a	×	b	×	N/A
14	Deniable	Definite	73	M	N/A	4	a	a	N/A	N/A	×	N/A
15	Deniable	Definite	72	F	249	Unclassified	a	b	○	N/A	×	○
16	Deniable	Definite	64	M	N/A	1	a	b	N/A	N/A	×	N/A
17	Deniable	Definite	60	M	15	1	a	b	×	b	×	N/A
18	Deniable	Definite	67	F	N/A	1	a	b	N/A	N/A	×	N/A
19	Deniable	Definite	66	M	170 (PO)	2	a	b	N/A	N/A	×	N/A
20	Deniable	Definite	70	M	N/A	1	a	b	N/A	N/A	×	N/A
21	Deniable	Definite	59	F	99	3	a	b	×	N/A	×	N/A
22	Deniable	Definite	68	M	185	1	a	b	○	N/A	×	N/A
23	Deniable	Definite	64	M	27.7	1	a	b	×	b	×	N/A
24	Deniable	Definite	81	M	55.4	1	a	a	×	b	×	N/A
25	Deniable	Definite	57	M	N/A	3	a	b	N/A	N/A	×	N/A
26	Deniable	Definite	77	M	231 (PO)	2	a	b	N/A	N/A	×	N/A
27	Deniable	Definite	82	M	76 (PO)	1	a	a	N/A	N/A	×	N/A
28	Deniable	Deniable	65	M	30	3	a	b	×	b	a	N/A

Abbreviations: IgG4‐SC2020, clinical diagnostic criteria for IgG4‐SC 2020; N/A, not applicable; PO, postoperative.

## 4 Discussion

We here validated the diagnostic performance of IgG4‐SC2020 in a multicenter study in Japan. Its sensitivity was significantly higher than that of IgG4‐SC2012 (99.0% vs. 89.1%, *p* < 0.001), while specificities for mimickers such as PC, PSC, and CCA showed no significant differences. However, four PSC cases were misclassified as probable IgG4‐SC by IgG4‐SC2020.

A key factor in the improved sensitivity was the incorporation of MRCP, which led to upgraded diagnosis in 97 patients (9.4%). These cases often showed elevated serum IgG4 levels, type 1 cholangiograms, and association of AIP. While endoscopic retrograde cholangiopancreatography (ERCP) was previously employed for diagnosing AIP and IgG4‐SC in Japan, it carries a risk of serious adverse events such as post‐ERCP pancreatitis [15, 16]. MRCP has become the preferred imaging modality for evaluating both the pancreatogram and cholangiogram in the diagnosis of AIP and IgG4‐SC in Japan, replacing ERCP due to its non‐invasiveness and improved image quality [17, 18]. It is now included in both JPS2018 for AIP and IgG4‐SC2020. The Japanese diagnostic criteria for PSC (2024) also recommend MRCP as the first‐line imaging modality [19], further highlighting its diagnostic importance.

Another contributing factor was the inclusion of non‐neoplastic cells in pathological findings. In 15 patients (1.5%), diagnoses were upgraded based on the “(v) No neoplastic cells identified”. This was particularly helpful in IgG4‐SC cases without AIP, as all of the 15 patients had no AIP. However, histological diagnosis using bile duct biopsy is challenging because typical IgG4‐SC features – lymphoplasmacytic infiltration and fibrosis‐ are located beneath the normal bile duct epithelium in the subepithelial stroma [20]. According to a nationwide Japanese survey [6], the rates of lymphoplasmacytic infiltration, fibrosis, > 10 IgG4‐positive plasma cells per high‐power field, storiform fibrosis, and obliterative phlebitis in bile duct biopsy samples were 32.9%, 16.9%, 0.6%, and 0.0%, respectively. Because histological features of IgG4‐SC are difficult to detect in bile duct biopsies, the primary role of biopsy remains the exclusion of malignancy such as CCA [6]. In the present cohort of 147 patients with CCA, the sensitivity of combined bile duct biopsy and cytology for detecting malignancy was 68.8% (95/138). However, according to a systematic review and meta‐analysis, the pooled sensitivity of bile duct biopsy in detecting malignant biliary strictures was 48.1% [21]. It should be noted that complete exclusion of malignancy is challenging, as the diagnostic yield of bile duct biopsy for detecting malignancy remains limited.

Kidney lesions, newly included as “other organ involvement,” led to diagnostic upgrades in only one patient, although IgG4‐related kidney disease was present in 98 patients (9.5%). The higher incidence compared to a 2019 nationwide study (4.9%) likely reflects increased clinical awareness following their incorporation into JPS2018 [12] and IgG4‐SC2020.

Conversely, diagnoses were downgraded in 10 patients under the IgG4‐SC2020 criteria. In three patients, bile duct wall thickening (II) could not be identified, despite two having type 1 and one having a type 2 cholangiogram. It is often difficult to detect thickening in strictures located in the intrapancreatic or intrahepatic bile ducts due to the surrounding pancreatic or hepatic parenchyma. Since bile duct wall thickening is newly incorporated in the IgG4‐SC2020, the criteria may be limited in such cases. The remaining seven patients had cholangiograms of type 3 (2 cases), type 4 (3 cases), or were unclassified (2 cases). Among them, two had normal serum IgG4 levels, and three were not associated with AIP. These features are not typical IgG4‐SC. Furthermore, EUS or IDUS was not performed in any of these cases, and bile duct biopsies were not conducted in four. Both IDUS and bile duct biopsy are useful in differentiating IgG4‐SC from PSC and CCA [22, 23, 24]. These procedures are essential for accurate diagnosis under the IgG4‐SC2020 criteria.

PC, PSC, and CCA are major mimickers of IgG4‐SC, and high specificity of the diagnostic criteria for IgG4‐SC is essential. Clinical features such as sex, age, and serum IgG4 levels differed between IgG4‐SC and these mimickers. While specificity remained 100% for PC and CCA, it dropped slightly for PSC (97.5%). All four were misdiagnosed as probable IgG4‐SC based on a combination of ERC (Ia), EUS/IDUS (IIa), elevated serum IgG4 (III), and absence of neoplastic cells on pathology (IVb). However, since PSC is a benign disease, the “non‐neoplastic” pathology criterion lacks discriminatory power. Cholangiographic and IDUS findings differ between IgG4‐SC and PSC, reflecting distinct pathological processes [23, 24, 25]. Therefore, careful interpretation of ERC and IDUS may be warranted when type 2 cholangiogram, elevated IgG4, and non‐neoplastic biopsy findings are present. Importantly, IgG4‐SC2020 maintained 100% specificity for both PC and CCA, supporting its utility in distinguishing IgG4‐SC from malignancy.

The preoperative sensitivity of IgG4‐SC2020 decreased from 99.0% to 96.8% when evaluated in all 1034 patients with IgG4‐SC, including the subset of 28 patients who underwent surgery for suspected malignancy and postoperatively confirmed as IgG4‐SC. In this subset of 28 patients, the preoperative sensitivity of IgG4‐SC2020 was 14.3%. These patients often had type 1 or 4 cholangiograms and normal serum IgG4. However, many lacked serum IgG4 measurements (39.2%) or biopsy results (52.9%). These omissions highlight the need for comprehensive diagnostic evaluation, including steroid trials as recommended in IgG4‐SC2020. Kubota et al. previously reported that IgG4‐SC2020 could help avoid unnecessary surgeries in patients with isolated hilar IgG4‐SC [26]. Type 4 IgG4‐SC (isolated hilar type) is often mistaken for hilar CCA. However, in the present study, eight patients with isolated type 1 IgG4‐SC and three with type 1 IgG4‐SC plus AIP underwent surgery. Clinicians should consider IgG4‐SC not only in cases with type 3 or 4 cholangiograms but also with type 1. According to a Japanese nationwide study [6], type 1 cholangiograms accounted for 23.8% of isolated IgG4‐SC cases—the second most common type after type 4 (30.9%)—despite isolated type 1 IgG4‐SC being regarded as rare [27]. Diagnostic efforts, including steroid trials recommended by IgG4‐SC2020, should be employed to avoid unnecessary surgeries.

This study has limitations. It was retrospective and based on physician‐reported questionnaires, and evaluating all diagnostic elements in detail was difficult due to the large sample size. Nonetheless, the nationwide multicenter design is a key strength.

In conclusion, this study is the first to validate IgG4‐SC2020 using a large Japanese multicenter cohort. Our findings demonstrate improved diagnostic performance of IgG4‐SC2020 compared to IgG4‐SC2012.

## Funding

This study was financially supported by Health and Labour Sciences Research Grants for the Study of Intractable Diseases from the Ministry of Health, Labor and Welfare, Japan (23FC1015).

## Conflicts of Interest

The authors declare no conflicts of interest.

## Supporting information


**Figure S1:** Change in pre and postoperative diagnosis according to the IgG4‐SC 2020 criteria among 28 patients underwent surgery.

**Figure S2:** Change in pre and postoperative diagnosis according to the IgG4‐SC 2012 criteria among 28 patients underwent surgery.

**Table S1:** Clinical Diagnostic Criteria of IgG4‐related sclerosing cholangitis 2012.

**Table S2:** Clinical Diagnostic Criteria of IgG4‐related sclerosing cholangitis 2020.

**Table S3:** Pre‐ and post‐operative diagnostic performance of IgG4‐SC2020 in 28 patients with IgG4‐SC underwent surgery.

## Data Availability

The data that support the findings of this study are available from the corresponding author upon reasonable request.

## References

[1] T. Kamisawa , T. Nakazawa , S. Tazuma , et al., “Clinical Practice Guidelines for IgG4‐Related Sclerosing Cholangitis,” Journal of Hepato‐Biliary‐Pancreatic Sciences 26 (2019): 9–42. 30575336 10.1002/jhbp.596 PMC6590186

[2] T. Nakazawa , T. Kamisawa , K. Okazaki , et al., “Clinical Diagnostic Criteria for IgG4‐Related Sclerosing Cholangitis 2020: (Revision of the Clinical Diagnostic Criteria for IgG4‐Related Sclerosing Cholangitis 2012),” Journal of Hepato‐Biliary‐Pancreatic Sciences 28 (2021): 235–242. 33586343 10.1002/jhbp.913

[3] T. Kamisawa , N. Funata , Y. Hayashi , et al., “A New Clinicopathological Entity of IgG4‐Related Autoimmune Disease,” Journal of Gastroenterology 38 (2003): 982–984. 14614606 10.1007/s00535-003-1175-y

[4] T. Kamisawa , Y. Zen , S. Pillai , and J. H. Stone , “IgG4‐Related Disease,” Lancet (London, England) 385 (2015): 1460–1471. 25481618 10.1016/S0140-6736(14)60720-0

[5] A. Tanaka , M. Mori , K. Kubota , et al., “Epidemiological Features of Immunoglobulin G4‐Related Sclerosing Cholangitis in Japan,” Journal of Hepato‐Biliary‐Pancreatic Sciences 27 (2020): 598–603. 32603554 10.1002/jhbp.793

[6] I. Naitoh , T. Kamisawa , A. Tanaka , et al., “Clinical Characteristics of Immunoglobulin IgG4‐Related Sclerosing Cholangitis: Comparison of Cases With and Without Autoimmune Pancreatitis in a Large Cohort,” Digestive and Liver Disease 53 (2021): 1308–1314. 33664004 10.1016/j.dld.2021.02.009

[7] K. Kubota , T. Kamisawa , T. Nakazawa , et al., “Steroid Therapy Still Plays a Crucial Role and Could Serve as a Bridge to the Next Promising Treatments in Patients With IgG4‐Related Sclerosing Cholangitis: Results of a Japanese Nationwide Study,” Journal of Hepato‐Biliary‐Pancreatic Sciences 29 (2022): 884–897, 10.1002/jhbp.1157. 35460190

[8] K. Kubota , T. Kamisawa , T. Nakazawa , et al., “Reducing Relapse Through Maintenance Steroid Treatment Can Decrease the Cancer Risk in Patients With IgG4‐Sclerosing Cholangitis: Based on a Japanese Nationwide Study,” Journal of Gastroenterology and Hepatology 38 (2023): 556–564. 36403136 10.1111/jgh.16066

[9] A. Ghazale , S. T. Chari , L. Zhang , et al., “Immunoglobulin G4‐Associated Cholangitis: Clinical Profile and Response to Therapy,” Gastroenterology 134 (2008): 706–715. 18222442 10.1053/j.gastro.2007.12.009

[10] H. Ohara , K. Okazaki , H. Tsubouchi , et al., “Clinical Diagnostic Criteria of IgG4‐Related Sclerosing Cholangitis 2012,” Journal of Hepato‐Biliary‐Pancreatic Sciences 19 (2012): 536–542. 22717980 10.1007/s00534-012-0521-y

[11] T. Shimosegawa , S. T. Chari , L. Frulloni , et al., “International Consensus Diagnostic Criteria for Autoimmune Pancreatitis: Guidelines of the International Association of Pancreatology,” Pancreas 40 (2011): 352–358. 21412117 10.1097/MPA.0b013e3182142fd2

[12] S. Kawa , T. Kamisawa , K. Notohara , et al., “Japanese Clinical Diagnostic Criteria for Autoimmune Pancreatitis, 2018: Revision of Japanese Clinical Diagnostic Criteria for Autoimmune Pancreatitis, 2011,” Pancreas 49 (2020): e13. 31856100 10.1097/MPA.0000000000001443 PMC6946098

[13] T. Nakazawa , K. Notohara , S. Tazuma , et al., “The 2016 Diagnostic Criteria for Primary Sclerosing Cholangitis,” Journal of Gastroenterology 52 (2017): 838–844. 27921168 10.1007/s00535-016-1286-x

[14] C. J. Clopper and E. S. Pearson , “The Use of Confidence or Fiducial Limits Illustrated in the Case of the Binomial,” Biometrika 26 (1934): 404–413.

[15] V. S. Akshintala , K. Kanthasamy , F. A. Bhullar , et al., “Incidence, Severity, and Mortality of Post‐ERCP Pancreatitis: An Updated Systematic Review and Meta‐Analysis of 145 Randomized Controlled Trials,” Gastrointestinal Endoscopy 98 (2023): 1–6.e12. 37004815 10.1016/j.gie.2023.03.023

[16] S. Mukai , Y. Takeyama , T. Itoi , et al., “Clinical Practice Guidelines for Post‐ERCP Pancreatitis 2023,” Digestive Endoscopy 37 (2025): 573–587. 40132896 10.1111/den.15004

[17] S. Yanagisawa , Y. Fujinaga , T. Watanabe , et al., “Usefulness of Three‐Dimensional Magnetic Resonance Cholangiopancreatography With Partial Maximum Intensity Projection for Diagnosing Autoimmune Pancreatitis,” Pancreatology 17 (2017): 567–571. 28506431 10.1016/j.pan.2017.05.001

[18] M. Takahashi , Y. Fujinaga , K. Notohara , et al., “Diagnostic Imaging Guide for Autoimmune Pancreatitis,” Japanese Journal of Radiology 38 (2020): 591–612. 32297064 10.1007/s11604-020-00971-z

[19] I. Naitoh , H. Isayama , N. Akamatsu , et al., “The 2024 Diagnostic Criteria for Primary Sclerosing Cholangitis,” Journal of Gastroenterology 60 (2025): 1221–1231. 40504416 10.1007/s00535-025-02265-5 PMC12450801

[20] K. Notohara , “Histological Features of Autoimmune Pancreatitis and IgG4‐Related Sclerosing Cholangitis With a Correlation With Imaging Findings,” Journal of Medical Ultrasonics (2001) 48 (2021): 581–594. 34669070 10.1007/s10396-021-01148-5

[21] U. Navaneethan , B. Njei , P. G. Venkatesh , J. J. Vargo , and M. A. Parsi , “Fluorescence In Situ Hybridization for Diagnosis of Cholangiocarcinoma in Primary Sclerosing Cholangitis: A Systematic Review and Meta‐Analysis,” Gastrointestinal Endoscopy 79 (2014): 943–950. 24360654 10.1016/j.gie.2013.11.001

[22] I. Naitoh , T. Nakazawa , H. Ohara , et al., “Endoscopic Transpapillary Intraductal Ultrasonography and Biopsy in the Diagnosis of IgG4‐Related Sclerosing Cholangitis,” Journal of Gastroenterology 44 (2009): 1147–1155. 19636664 10.1007/s00535-009-0108-9

[23] K. Kubota , S. Kato , T. Uchiyama , et al., “Discrimination Between Sclerosing Cholangitis‐Associated Autoimmune Pancreatitis and Primary Sclerosing Cholangitis, Cancer Using Intraductal Ultrasonography,” Digestive Endoscopy 23 (2011): 10–16. 21198911 10.1111/j.1443-1661.2010.01039.x

[24] I. Naitoh , T. Nakazawa , K. Hayashi , et al., “Comparison of Intraductal Ultrasonography Findings Between Primary Sclerosing Cholangitis and IgG4‐Related Sclerosing Cholangitis,” Journal of Gastroenterology and Hepatology 30 (2015): 1104–1109. 25594435 10.1111/jgh.12894

[25] T. Nakazawa , H. Ohara , H. Sano , et al., “Cholangiography Can Discriminate Sclerosing Cholangitis With Autoimmune Pancreatitis From Primary Sclerosing Cholangitis,” Gastrointestinal Endoscopy 60 (2004): 937–944. 15605009 10.1016/s0016-5107(04)02229-1

[26] K. Kubota , E. Iwasaki , T. Ishikawa , et al., “Diagnosis of Isolated Hilar‐/Extrahepatic‐Type IgG‐4‐Related Sclerosing Cholangitis Can Be Increased by Improved Recognition of This Condition‐A Japanese Multicenter Analysis,” Journal of Hepato‐Biliary‐Pancreatic Sciences 31 (2024): 647–657. 39123289 10.1002/jhbp.12053

[27] T. Nakazawa , Y. Ikeda , Y. Kawaguchi , et al., “Isolated Intrapancreatic IgG4‐Related Sclerosing Cholangitis,” World Journal of Gastroenterology 21 (2015): 1334–1343. 25632210 10.3748/wjg.v21.i4.1334 PMC4306181

