# Supplementary figures and images for: Multicenter Validation Study of the Clinical Diagnostic Criteria for IgG4‐Related Sclerosing Cholangitis 2020 in Japan
Source: J Hepatobiliary Pancreat Sci. 2026 Jan 7;33(4):294–303. doi: 10.1002/jhbp.70056 (PMC13113202; doi:10.1002/jhbp.70056)

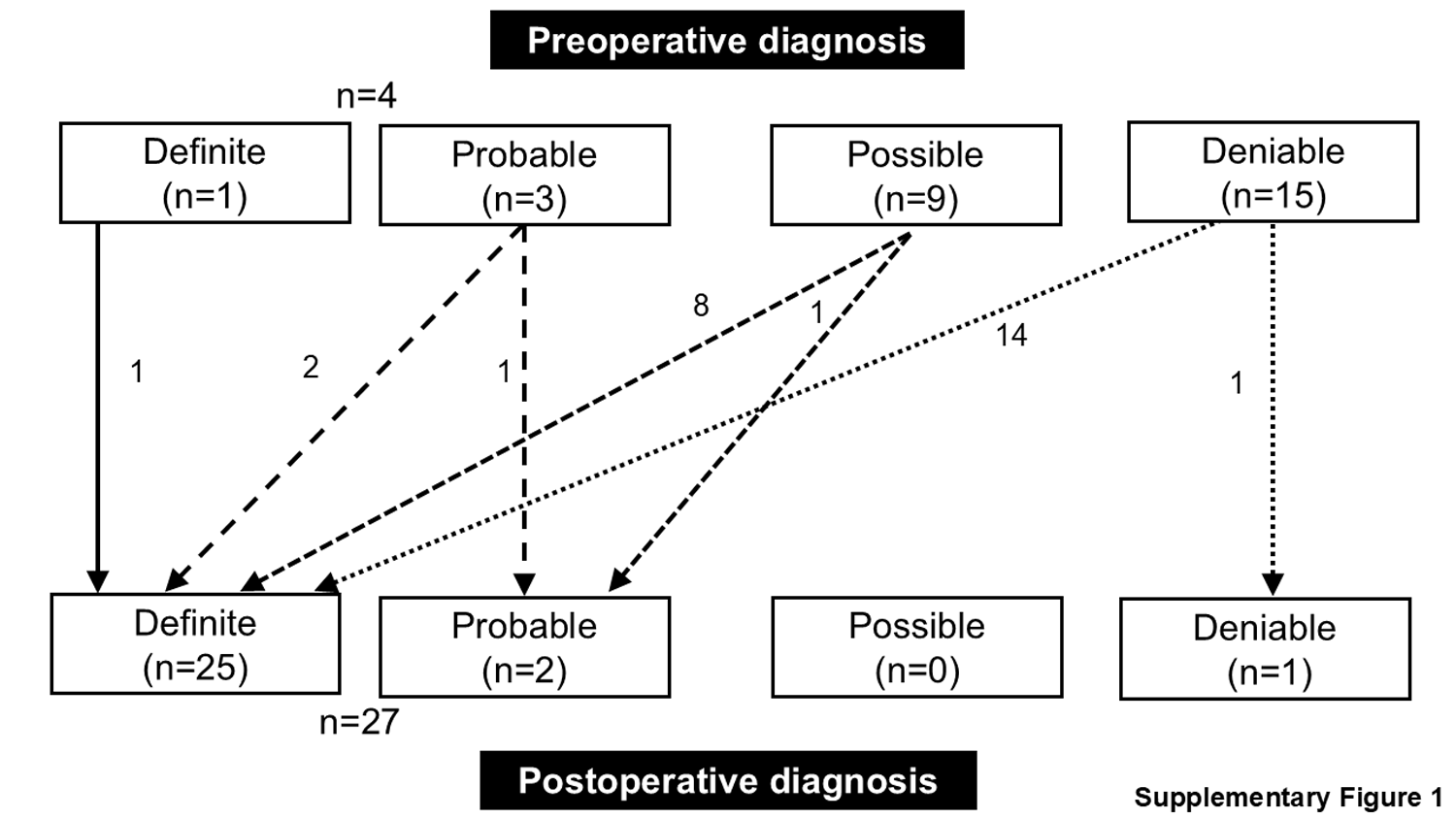

Supplement: Supplementary file 1 — Figure S1: Change in pre and postoperative diagnosis according to the IgG4‐SC 2020 criteria among 28 patients underwent surgery. [file JHBP-33-294-s004.tif]

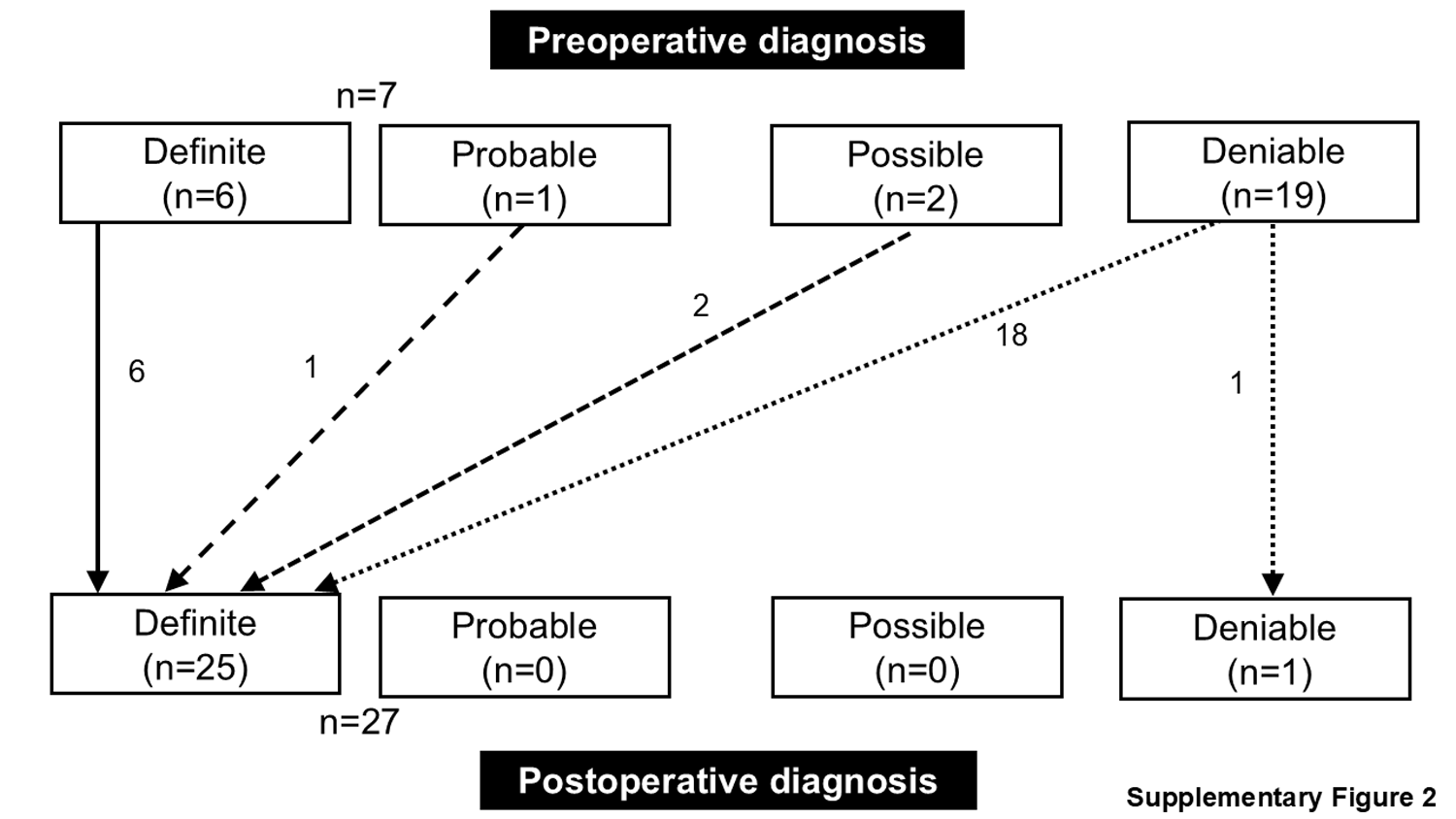

Supplement: Supplementary file 2 — Figure S2: Change in pre and postoperative diagnosis according to the IgG4‐SC 2012 criteria among 28 patients underwent surgery. [file JHBP-33-294-s002.tif]
