# Supplementary material for: Multicenter Validation Study of the Clinical Diagnostic Criteria for IgG4‐Related Sclerosing Cholangitis 2020 in Japan
Source: J Hepatobiliary Pancreat Sci. 2026 Jan 7;33(4):294–303. doi: 10.1002/jhbp.70056 (PMC13113202; doi:10.1002/jhbp.70056)
Supplement: Supplementary file 3 — Table S1: Clinical Diagnostic Criteria of IgG4‐related sclerosing cholangitis 2012. [file JHBP-33-294-s005.docx]

| **Supplementary Table1. Clinical Diagnostic Criteria of IgG4-related sclerosing cholangitis 2012**  **Adapted from ref [10], 2012, with permission from John Wiley and Sons.** | | | |
| --- | --- | --- | --- |
| Diagnostic items | | | |
|  | (1) Biliary tract imaging reveals diffuse or segmental narrowing of the intrahepatic and/or extrahepatic bile duct associated with the thickening of bile duct wall | | |
|  | (2) Hematological examination shows elevated serum IgG4 concentrations (≥135mg/dl) | | |
|  | (3) Coexistence of autoimmune pancreatitis, IgG4-related dacryoadenitis/sialadenitis, or IgG4-related retroperitoneal fibrosis | | |
|  | (4) Histopathologic examination shows: | | |
|  |  | a. Marked lymphocytic and plasmacyte infiltration and fibrosis. | |
|  |  | b. Infiltration of IgG4-positive plasma cells: >10 IgG4-positive plasma cells/HPF | |
|  |  | c. Storiform fibrosis | |
|  |  | d. Obliterative phlebitis | |
|  | Option: Effectiveness of steroid therapy | | |
|  |  | | |
| Diagnosis | | | |
|  | Definite: | | (1) + (3); (1) + (2) + (4) a, b; (1) + (4) a, b, c; (1) + (4) a, b, d |
|  | Probable: | | (1) + (2) + Option |
|  | Possible: | | (1) + (2) |
