# Supplementary material for: Multicenter Validation Study of the Clinical Diagnostic Criteria for IgG4‐Related Sclerosing Cholangitis 2020 in Japan
Source: J Hepatobiliary Pancreat Sci. 2026 Jan 7;33(4):294–303. doi: 10.1002/jhbp.70056 (PMC13113202; doi:10.1002/jhbp.70056)
Supplement: Supplementary file 4 — Table S2: Clinical Diagnostic Criteria of IgG4‐related sclerosing cholangitis 2020. [file JHBP-33-294-s001.docx]

| **Supplementary Table2. Clinical Diagnostic Criteria of IgG4-related sclerosing cholangitis 2020**  **Adapted from ref [2], 2021, with permission from John Wiley and Sons.** | | | | | |
| --- | --- | --- | --- | --- | --- |
| Diagnostic items | | | | | |
|  | I. Narrowing of the intrahepatic and/or extrahepatic bile duct | | | | |
|  |  | a. ERC; b. MRCP | | | |
|  | II. Thickening of the bile-duct wall | | | | |
|  |  | a. EUS/IDUS; b. CT/MRI/US | | | |
|  | III. Serological findings | | | | |
|  |  | Elevated levels of serum IgG4 (≥ 135 mg/dL) | | | |
|  | IV. Pathological findings among i)-v) listed below | | | | |
|  |  | a. i), ii), and v) are observed | | | |
|  |  | b. v) is observed | | | |
|  |  | c. All of i), ii), and v) and either or both of iii) or iv) are observed | | | |
|  |  |  | | (i) Marked lymphoplasmacytic infiltration and fibrosis | |
|  |  |  | | (ii) More than 10 IgG4-positive plasma cells /HPF | |
|  |  |  | | (iii) Storiform fibrosis | |
|  |  |  | | (iv) Obliterative phlebitis | |
|  |  |  | | (v) No neoplastic cells identified | |
|  | V. Other organ involvement (OOI) | | | | |
|  |  | a. Type 1 autoimmune pancreatitis | | | |
|  |  | b. IgG4-related dacryoadenitis/sialadenitis (Mikulicz disease), IgG4-related retroperitoneal fibrosis, IgG4-related kidney lesion | | | |
|  | VI. Effectiveness of steroid therapy | | | | |
|  |  | | | | |
| Diagnosis | | | | | |
|  | Definite: | | | | |
|  | ① Va+ | | | | |
|  |  | | Cholangiographic classification Types 1, 2 | | Ia/b + IIa/b + III/VI |
|  |  | | Cholangiographic classification Types 3, 4 | | Ia + IIa + IVb + III/VI |
|  | ② Va+ | | | | |
|  |  | | Cholangiographic classification Types 1, 2, 3, 4 | | Ia + IIa + III + IVa/VI |
|  | ③ Pathological definite diagnosis | | | | Ⅳc |
|  | Probable: | | | |  |
|  | ① Va+ | | | | |
|  |  | | Cholangiographic classification Types 1, 2 | | Ia/b + IIa/b |
|  |  | | Cholangiographic classification Types 3, 4 | | Ia + IIa + IVb  Ia/b + IIb + VI |
|  | ② Va+ | | | | |
|  |  | | Cholangiographic classification Types 1, 2, 3, 4 | | Ia + IIa + IVa  Ia + IIa + III + IVb  Ib + IIa + III + VI |
|  | Possible | | | | |
|  | ① Va+ | | | | |
|  |  | | Cholangiographic classification Types 3, 4 | | Ia/b + IIa  Ib + IIb + III |
|  | ② Va+ | | | |  |
|  |  | | Cholangiographic classification Types 1, 2, 3, 4 | | Ia + IIa + III/Vb/VI  Ib + IIb + III + VI |
| CT, computed tomography; ERC, endoscopic ultrasonography; EUS, endoscopic ultrasonography; IDUS, intraductal ultrasonography; MRCP, magnetic resonance cholangiopancreatography; MRI, magnetic resonance imaging; US, ultrasonography | | | | | |
