# Supplementary material for: Multicenter Validation Study of the Clinical Diagnostic Criteria for IgG4‐Related Sclerosing Cholangitis 2020 in Japan
Source: J Hepatobiliary Pancreat Sci. 2026 Jan 7;33(4):294–303. doi: 10.1002/jhbp.70056 (PMC13113202; doi:10.1002/jhbp.70056)
Supplement: Supplementary file 5 — Table S3: Pre‐ and post‐operative diagnostic performance of IgG4‐SC2020 in 28 patients with IgG4‐SC underwent surgery. [file JHBP-33-294-s003.docx]

| **Supplementary Table 3. Pre- and post-operative diagnostic performance of IgG4-SC2020 in 28 patients with IgG4-SC underwent surgery** | | | | | | | | | |
| --- | --- | --- | --- | --- | --- | --- | --- | --- | --- |
| Disease | Pre-operation | | | |  | Post-operation | | | |
|  | Definite,  n (%) | Probable,  n (%) | Possible,  n (%) | Deniable,  n (%) |  | Definite,  n (%) | Probable,  n (%) | Possible,  n (%) | Deniable,  n (%) |
| IgG4-SC  (n=28) | 1 (3.6) | 3 (10.7) | 9 (32.1) | 15 (53.6) |  | 25 (89.2) | 2 (7.1) | 0 (0) | 1 (3.6) * |
|  | 4 (14.3) ** | | 24 (85.7) | |  | 27 (96.4) ** | | 1 (3.6) | |
| IgG4-SC, IgG4-realated sclerosing cholangitis  *This case can be diagnosed as definitive according to the IgG4-SC2012  **p < 0.001 between before and after surgery | | | | | | | | | |
